# Dietary intake and visceral adiposity in older adults: The Multiethnic Cohort Adiposity Phenotype study

**DOI:** 10.1002/osp4.734

**Published:** 2024-01-22

**Authors:** Melissa A. Merritt, Unhee Lim, Johanna W. Lampe, Tanyaporn Kaenkumchorn, Carol J. Boushey, Lynne R. Wilkens, John A. Shepherd, Thomas Ernst, Loïc Le Marchand

**Affiliations:** ^1^ The Daffodil Centre The University of Sydney, a Joint Venture with Cancer Council NSW Sydney New South Wales Australia; ^2^ Faculty of Medicine and Health The University of Sydney Sydney New South Wales Australia; ^3^ Cancer Epidemiology Program University of Hawaii Cancer Center University of Hawaii Honolulu Hawaii USA; ^4^ Division of Public Health Sciences Fred Hutchinson Cancer Research Center Seattle Washington USA; ^5^ Division of Pediatric Gastroenterology, Hepatology, and Nutrition Department of Pediatrics Seattle Children's Hospital Seattle Washington USA; ^6^ Department of Diagnostic Radiology and Nuclear Medicine University of Maryland Baltimore Maryland USA

**Keywords:** adiposity, diet, ethnic minorities, visceral fat

## Abstract

**Background:**

There are established links between the accumulation of body fat as visceral adipose tissue (VAT) and the risk of developing obesity‐associated metabolic disease. Previous studies have suggested that levels of intake of specific foods and nutrients are associated with VAT accumulation after accounting for total energy intake.

**Objective:**

This study assessed associations between a priori selected dietary factors on VAT quantified using abdominal magnetic resonance imaging.

**Methods:**

The cross‐sectional Multiethnic Cohort Adiposity Phenotype Study included *n* = 395 White, *n* = 274 Black, *n* = 269 Native Hawaiian, *n* = 425 Japanese American and *n* = 358 Latino participants (mean age = 69 years ± 3 SD). Participants were enrolled stratified on sex, race, ethnicity and body mass index. General linear models were used to estimate the mean VAT area (cm^2^) for participants categorized into quartiles based on their dietary intake of selected foods/nutrients adjusting for age, sex, racial and ethnic groups, the total percentage fat from whole‐body dual energy X‐ray absorptiometry and total energy.

**Results:**

There were significant inverse associations with VAT for dietary intake of total vegetables, total fruits (including juice), cereals, whole grains, calcium, copper and dietary fiber (*p*‐trend ≤0.04). Positive trends were observed for VAT for participants who reported higher intake of potatoes, total fat and saturated fatty acids (SFA) (*p*‐trend ≤0.02). Foods/nutrients that met the multiple testing significance threshold were total fruits, whole grains, copper, dietary fiber and SFA intake.

**Conclusions:**

These results highlight foods and nutrients including SFA, total fruit, whole grains, fiber and copper as potential candidates for future research to inform dietary guidelines for the prevention of chronic disease among older adults.

## 1 INTRODUCTION

Anthropometric measures are used to predict a person's health status. Typically, body mass index (BMI; kg/m^2^), a measure of general adiposity, is used to measure excess body fat in clinical practice; however, this measure is limited because it does not capture body fat distribution or distinguish fat from lean mass, such as muscle. The relative size of body fat deposits, particularly visceral adipose tissue (VAT), which is most often measured using computed tomography or magnetic resonance imaging (MRI), is a better indicator than BMI of obesity‐associated metabolic stress.^1^ Furthermore, higher VAT levels are correlated with a variety of metabolic abnormalities and diseases including insulin resistance and type 2 diabetes, atherogenic dyslipidemia and cardiovascular disease.^2^

Little is known about associations between the intake of specific dietary factors and the propensity to accumulate VAT. Fischer et al.^3^ conducted a systematic review to summarize evidence from human observational and controlled intervention studies that evaluated associations between qualitative aspects of diet and associations with VAT and/or subcutaneous adipose tissue (SAT). They suggested that intakes of specific foods and nutrients (e.g., dietary fiber intake) predominantly influence VAT accumulation after accounting for total energy intake, whereas SAT seems to be determined more by excess total energy intake.^3^, ^4^ Foods and nutrients that were linked with VAT accumulation in the systematic review included positive associations for alcohol, sugar‐sweetened beverages, refined grain foods, high glycemic load and total fat; inverse associations were observed for whole grain foods, coffee, dietary fiber, calcium and protein.^3^

Since the time of publication of the Fischer et al.^3^ review, four additional cross‐sectional studies on dietary intake in relation to VAT accumulation were identified; three of the studies evaluated two independent European populations of approximately *n* = 580 participants^4^, ^5^ and *n* = 6671 participants^6^; the Tayyem et al.^7^ study included *n* = 167 participants from Jordan. These studies observed positive associations with VAT for participants who reported a higher intake of potatoes^5^ and inverse associations with VAT were reported for higher intakes of total fruit, total vegetables, margarine^6^ and cereals.^5^ Both positive and negative associations with VAT have been reported for dietary intake of copper^4^, ^7^ and total fat.^3^, ^7^ Tayyem et al.^7^ also reported inverse associations with monounsaturated, omega‐3 and omega‐6 fatty acids on VAT. A recent clinical trial investigated the association between overfeeding saturated fatty acids (SFAs) versus polyunsaturated fatty acids (specifically, omega‐6) on VAT storage among *N* = 39 young Swedish adults (BMI 18–27 kg/m^2^) and they observed that SFAs caused a 2‐fold higher increase in VAT as compared with the omega‐6 group.^8^

Previous studies included mostly White participants. To better understand how dietary intake of specific foods and nutrients may influence VAT accumulation, additional studies are needed to assess dietary intake in relation to VAT accumulation in racially and ethnically diverse study populations. This is critical because results from the Multiethnic Cohort Adiposity Phenotype Study (MEC‐APS) have highlighted racial and ethnic differences in the propensity to accumulate VAT; specifically, relative amounts of VAT were highest in Japanese Americans, moderate in Whites, Latinos, and Native Hawaiians, and lowest in African American participants.^9^ An earlier analysis in the MEC‐APS has also demonstrated that a higher diet quality (assessed using the healthy eating index, HEI‐2010) was associated with lower adiposity values for both men and women.^10^ Data are needed from diverse study populations to support a “Precision Nutrition” approach that accounts for racial and ethnic diversity as well as related factors (genetics, microbiome, food environment, etc.) in the development of targeted nutrition guidelines.^11^ The current study objective was to investigate associations between a priori selected dietary factors (identified in the previous literature) with VAT, adjusting for total adiposity and other factors, using a cross‐sectional study design in the MEC‐APS.

## 2 METHODS

### 2.1 Study population

The MEC included approximately *n* = 215,000 men and women who were 45–75 years of age at baseline (1993–1996).^12^ The MEC includes five major racial and ethnic groups based on self‐reported race and ethnicity: Japanese American, Native Hawaiian and White (mostly from Hawaii); African American and Latino (mostly from Los Angeles County, California). In 2012, a subset of surviving MEC participants aged 58–74 years were selected for the Adiposity Phenotype Study (MEC‐APS).^9^ In brief, individuals who self‐reported only one race were prioritized, except for Native Hawaiians who could be part‐Hawaiian. Exclusion criteria included reported BMI outside 18.5–40 kg/m^2^; history of smoking in the previous 2 years; soft or metal body implants or amputation; insulin or thyroid medications; and serious medical conditions (e.g., dialysis, chronic hepatitis). Participants were enrolled stratified on sex, race and ethnicity and six BMI categories (18.5–21.9, 22–24.9, 25–26.9, 27–29.9, 30–34.9 and 35–40 kg/m^2^, based on self‐reported weight and height) in order to balance the sample size across the BMI distribution and all sex/ethnic groups for optimal comparability. The study was conducted at the University of Hawaii and at the University of Southern California. Institutional Review Boards at both institutions approved the study protocol. All participants signed an informed consent form.

The overall participation rate was 15% among the *n* = 12,602 individuals invited, and 23% after excluding participants who were willing to participate but were ineligible; *n* = 1861 individuals remained for the present study (Figure S1). The participation rate included individuals who were not interested (had declined) in the denominator since the recruitment was from a closed population from the MEC. These participants underwent a clinic visit, which involved anthropometric measurements, fasting blood sample collection, whole‐body dual energy X‐ray absorptiometry (DXA) for body composition, abdominal MRI for VAT quantification and study questionnaires including a quantitative food frequency questionnaire (FFQ).

### 2.2 Imaging

Differences in body fat distribution were estimated using DXA for the whole body, trunk, arms and legs, from which skeletal muscle mass was derived.^13^ An abdominal MRI scan was acquired on 3‐T scanners to quantify VAT areas (cm^2^) at four lumbar cross‐sections (L1–L5) using an axial gradient‐echo sequence with breath holds and to calculate percentage fat in the liver using a series of axial triple gradient‐echo Dixon‐type scans.^14^ MRI measures were calibration adjusted for minimal differences between the scanners at the two study sites based on *n* = 15 healthy volunteers who were scanned at both sites.

### 2.3 Dietary assessment

Participants completed a self‐administered 20‐page FFQ at their MEC‐APS clinic visit that queried intakes for over 180 food items, as well as questions on demographics, medical conditions, anthropometric measures, physical activity and other lifestyle factors.^12^, ^15^ The FFQ was developed for the diverse racial and ethnic populations in MEC and it has been validated in a calibration study using three 24‐h food recalls, which showed mostly high correlations between the FFQ and the 24‐h recalls.^15^ For example, correlations for energy‐adjusted nutrient intakes were calculated separately from racial and ethnic groups and ranged as follows for males and females, respectively: calcium, 0.68–0.80 and 0.52–0.68; carbohydrate, 0.41–0.54 and 0.20–0.73; fat (total), 0.53–0.65 and 0.34–0.68; fat (saturated), 0.57–0.69 and 0.41–0.76; fiber, 0.68–0.80 and 0.60–0.73; protein, 0.25–0.39 and 0.27–0.56.

In the current study, a priori specified foods and nutrients were selected from the literature that were associated with the propensity to accumulate VAT and for which comparable data on dietary assessment were available in the MEC‐APS. The PubMed database was searched with a focus on meta‐analyses, randomized controlled studies, prospective cohort studies, large cross‐sectional studies and reviews for recent studies that identified dietary factors that were statistically significantly associated with VAT (from September 2013 through 31 July 2021, focusing on new studies that were published after the Fischer et al.^3^ review) (Supplemental Methods, Table S1).

### 2.4 Statistical methods

For the present cross‐sectional study focusing on diet and VAT, the following additional participants were excluded due to: DXA scans yielding invalid estimates (previously unreported joint replacements and other implants; *n* = 21), invalid MRI scans to measure VAT (*n* = 59) or liver fat (*n* = 24) and missing information on diet at the MEC‐APS clinic visit (*n* = 36), leaving *n* = 1721 participants in the study.

General linear models were used to estimate the multivariable‐adjusted mean VAT area (cm^2^) for participants categorized into quartiles based on their reported dietary intake of specific foods and nutrients. Quartiles were presented to illustrate differences in the mean VAT area across low, medium and high levels of dietary intake. Multivariable models were adjusted for factors that influence VAT: age (continuous), sex (male [ref], female), racial and ethnic groups (White [ref], African American, Native Hawaiian, Japanese, Latino), total adiposity from DXA (continuous, to accurately determine relative regional adiposity), and total energy (kilocalories per day, continuous). We tested whether additional adjustment for either education or physical activity changed the risk estimates and the results were very similar; therefore, these factors were not included in the final multivariable adjusted models. Sensitivity analyses additionally adjusting for total fat mass (kg, continuous) were conducted to assess changes in VAT when overall adiposity was constant. Data on dietary intake and adjustment variables were complete (there were no missing data).

*p*‐values for trend (*p*‐trend) were calculated using a variable that was assigned the median value for each quantile. A two‐tailed *p* < 0.05 was considered statistically significant. To account for multiple testing, a Bonferroni correction was used (i.e., calculation of a critical value = 0.05/number of hypotheses tested) to highlight selected foods and nutrients that were statistically significant using conservative criteria. It was determined a priori not to stratify dietary analyses by race and ethnicity because of the limited sample size for each racial and ethnic group. Analyses were conducted using *R* version 4.1.0.^16^

## 3 RESULTS

The overall sample included *n* = 1721 MEC‐APS participants with a mean of 69 ± 3 (SD) years of age (Table 1). Male and female participants were equally represented and the race and ethnicity distribution was 24.7% Japanese American, 23.0% White, 20.8% Latino, 15.9% Black and 15.6% Native Hawaiian. In comparisons of the population characteristics of MEC‐APS participants classified by quartiles of VAT area, there were differences by sex; the highest/fourth quartile (Q4) of VAT included a high proportion of men (VAT Q4, 80.7% male vs. Q1, 28.6% male). The examination of racial and ethnic groups by VAT quartiles showed that a large proportion of Latino participants had high VAT levels (Q4 of VAT included 30.2% Latino, 21.9% Japanese American, 21.2% White, 15.8% Native Hawaiian and 10.9% African American participants). As expected with increasing VAT, there were higher levels of total energy intake (Q4 of VAT, mean total energy = 1998 kcal/day vs. Q1, 1715) and higher BMI (Q4 of VAT, mean BMI = 31.4 kg/m^2^ vs. Q1, 23.6). Participants with high VAT were also more likely to be former smokers (VAT Q4, 51.9% former smokers vs. Q1, 32.3%) (current smoking was a study exclusion criteria). The following characteristics did not differ by VAT quartiles: physical activity level, education level and total fat percentage (assessed by DXA).

**TABLE 1:** Characteristics of participants (*N* = 1721) according to visceral fat in the Multiethnic Cohort Adiposity Phenotype Study.

	Visceral fat area (increasing quartiles)				
	Overall	Q1 (*n* = 430)	Q2 (*n* = 430)	Q3 (*n* = 431)	Q4 (*n* = 430)
Means (SD)					
Age at clinic visit, years	69.2 (2.7)	68.9 (2.8)	69.2 (2.7)	69.1 (2.7)	69.5 (2.6)
Physical activity, METS/day^a^	1.7 (0.3)	1.7 (0.3)	1.7 (0.3)	1.6 (0.3)	1.6 (0.3)
Total energy, kcal/day	1870 (919)	1715 (788)	1890 (943)	1877 (818)	1998 (1080)
Maximum education, years^a^	14.8 (2.7)	15.4 (2.4)	14.9 (2.7)	14.6 (2.8)	14.4 (2.9)
Body mass index, kg/m^2^	27.8 (4.7)	23.6 (3.3)	27.3 (3.8)	28.9 (4.0)	31.4 (4.0)
Total fat (DXA), %	33.5 (7.8)	31.7 (8.1)	35.1 (8.0)	34.0 (8.3)	33.1 (6.1)
Visceral fat area (MRI), cm^2^	167.6 (83.3)	73.0 (21.8)	131.4 (14.4)	185.8 (17.1)	280.3 (58.9)
Percentages					
Male, %	49.2	28.6	34.7	52.7	80.7
Female, %	50.8	71.4	65.3	47.3	19.3
Race and ethnicity					
White, %	23.0	28.4	22.3	20.0	21.2
Black, %	15.9	20.5	19.1	13.2	10.9
Native Hawaiian, %	15.6	15.1	15.6	16.0	15.8
Japanese American, %	24.7	25.1	24.0	27.8	21.9
Latino, %	20.8	10.9	19.1	23.0	30.2
Location					
Hawaii, %	63.3	68.8	61.9	63.8	58.8
California, %	36.7	31.2	38.1	36.2	41.2
Smoking status					
Never smoker, %	60.7	67.7	64.9	62.2	48.1
Former smoker, %	39.3	32.3	35.1	37.8	51.9

Abbreviations: DXA, whole‐body dual energy X‐ray absorptiometry; METs, metabolic equivalent of tasks; MRI, abdominal magnetic resonance imaging.

^a^ There were no missing data except for physical activity (3% missing) and education (0.6% missing).

Results of analyses focusing on a priori selected dietary factors are shown in Table 2. The *p*‐trend indicates the strength of a linear dose‐response association across quartiles of dietary intake. For the following foods and nutrients, significant positive associations with VAT accumulation were confirmed: white potatoes, total fat and SFAs (*p*‐trend ≤0.02). Additionally, significant inverse trends were observed (lower mean VAT area with higher quartiles of dietary intake) for total vegetables, total fruits (including juice), cereals, whole grains, calcium, copper and dietary fiber (*p*‐trend ≤0.04). Total vegetables and fruits assessed as cups/day are shown in Table S2. Inverse associations with margarine, monounsaturated and omega‐3 fatty acids on VAT were not confirmed and instead there were positive trends (*p*‐trend ≤0.04). The hypothesized positive association between carbohydrate intake and VAT was not observed in the current study; instead, there was an inverse trend of lower VAT for participants classified in higher quartiles of carbohydrate intake (*p*‐trend = 0.004). After accounting for multiple testing, the following dietary factors retained statistical significance: inverse associations for fruits, whole grains, copper and fiber; and the positive association for SFA intake with VAT area. In contrast, there was no significant trend between quartiles of dietary intake with mean VAT area for the following foods and nutrients: alcohol, coffee, sugar‐sweetened beverages, omega‐6 fatty acids and protein.

**TABLE 2:** Associations of intake of specific dietary factors with the propensity to accumulate visceral adipose tissue^a^ in the Multiethnic Cohort Adiposity Phenotype Study.

Variable	Quartiles of intake	Participants (*n*)	Median intake	Mean VAT area	VAT difference %	*p*‐trend^b^
				(95% CLs)^a^	(Q4 vs. Q1)	
Dietary variables hypothesized a priori to lower VAT						
Dietary fiber (G)	Q1	430	10.8	177.44 (170.86, 184.02)		
	Q2	430	17.1	172.70 (166.82, 178.58)		
	Q3	430	24.0	165.34 (159.57, 171.12)		
	Q4	431	36.2	152.27 (145.23, 159.32)	−7.6	<0.001*
Calcium (Mg)	Q1	430	379.3	173.88 (167.18, 180.57)		
	Q2	430	592.0	166.78 (160.90, 172.66)		
	Q3	431	820.7	166.06 (160.21, 171.91)		
	Q4	430	1218.1	160.73 (153.64, 167.82)	−3.9	0.03
Copper (Mg)^c^	Q1	430	0.8	179.38 (172.51, 186.25)		
	Q2	430	1.2	169.48 (163.52, 175.44)		
	Q3	431	1.6	166.82 (161.05, 172.58)		
	Q4	430	2.3	151.73 (144.08, 159.39)	−8.3	<0.001*
Protein (G)	Q1	430	39.6	166.39 (159.15, 173.63)		
	Q2	430	59.5	163.99 (157.94, 170.03)		
	Q3	431	78.7	169.02 (163.24, 174.80)		
	Q4	430	114.1	168.26 (160.03, 176.50)	0.56	0.60
Monounsaturated fatty acids (G)	Q1	430	13.5	160.38 (153.39, 167.37)		
	Q2	430	20.9	164.61 (158.56, 170.65)		
	Q3	431	28.6	169.71 (163.94, 175.48)		
	Q4	430	43.8	173.06 (165.16, 180.95)	3.8	0.04
Omega‐3 fatty acids (G)	Q1	430	0.8	162.34 (155.46, 169.22)		
	Q2	430	1.3	161.88 (155.90, 167.86)		
	Q3	431	1.7	169.96 (164.19, 175.74)		
	Q4	430	2.7	173.46 (165.85, 181.07)	3.3	0.03
Omega‐6 fatty acids (G)	Q1	430	6.9	169.12 (162.23, 176.02)		
	Q2	430	10.6	166.94 (160.94, 172.93)		
	Q3	431	15.0	164.13 (158.35, 169.92)		
	Q4	430	22.5	167.46 (159.82, 175.10)	−0.49	0.75
Total vegetables (G)	Q1	430	146.6	172.09 (165.78, 178.40)		
	Q2	430	257.9	167.12 (161.28, 172.96)		
	Q3	431	374.6	170.26 (164.49, 176.03)		
	Q4	430	627.2	158.19 (151.56, 164.82)	−4.2	0.01
All fruits plus juice (G)	Q1	430	66.4	172.58 (166.57, 178.58)		
	Q2	430	163.6	170.62 (164.81, 176.44)		
	Q3	431	285.2	167.04 (161.30, 172.79)		
	Q4	430	532.1	157.54 (151.35, 163.73)	−4.6	<0.001*
Breakfast cereals (G)	Q1	430	0.8	168.55 (162.69, 174.41)		
	Q2	430	3.2	171.98 (166.15, 177.81)		
	Q3	431	8.0	165.44 (159.69, 171.20)		
	Q4	430	22.7	161.80 (155.84, 167.76)	−2	0.03
Grains, whole (Oz)	Q1	430	0.4	175.75 (169.78, 181.73)		
	Q2	430	1.0	168.40 (162.64, 174.16)		
	Q3	431	1.8	164.71 (158.96, 170.46)		
	Q4	430	3.1	158.87 (152.86, 164.88)	−5	<0.001*
Margarine (G)	Q1	430	0.3	162.50 (156.50, 168.51)		
	Q2	430	1.1	164.97 (159.20, 170.74)		
	Q3	431	3.0	166.70 (160.92, 172.48)		
	Q4	430	7.0	173.42 (167.39, 179.46)	3.3	0.01
Total coffee (G)^d^	Q1	553	0	162.75 (157.63, 167.87)		
	Q2	344	8.8	167.41 (160.96, 173.86)		
	Q3	407	36.7	168.64 (162.71, 174.56)		
	Q4	417	229.8	170.43 (164.45, 176.41)	2.3	0.14
Dietary variables hypothesized a priori to increase VAT						
White potatoes (cup)	Q1	430	0	159.55 (153.51, 165.59)		
	Q2	430	0.1	165.01 (159.18, 170.83)		
	Q3	431	0.2	170.73 (164.97, 176.49)		
	Q4	430	0.4	172.26 (165.93, 178.59)	3.8	0.01
Carbohydrate (G)	Q1	430	117.8	177.44 (170.35, 184.54)		
	Q2	430	177.4	167.45 (161.41, 173.49)		
	Q3	431	233.1	164.59 (158.80, 170.38)		
	Q4	430	342.1	158.06 (150.05, 166.06)	−5.8	<0.01
Total fat (G)	Q1	430	35.7	162.19 (155.13, 169.24)		
	Q2	430	54.0	161.04 (155.00, 167.08)		
	Q3	431	74.0	169.96 (164.20, 175.73)		
	Q4	430	111.7	174.48 (166.49, 182.46)	3.7	0.02
Saturated fatty acids (G)	Q1	430	10.7	160.78 (153.90, 167.67)		
	Q2	430	16.2	163.05 (157.07, 169.02)		
	Q3	431	22.4	164.53 (158.75, 170.31)		
	Q4	430	34.8	179.31 (171.62, 187.00)	5.4	<0.01*
Sugar‐sweetened beverages (G)^d^	Q1	447	0	169.80 (164.05, 175.54)		
	Q2	426	3.7	164.54 (158.73, 170.35)		
	Q3	432	16.0	167.59 (161.84, 173.33)		
	Q4	416	73.9	165.61 (159.64, 171.59)	−1.2	0.61
Alcoholic beverages (*n* drinks)	Q1	430	0	165.65 (159.65, 171.64)		
	Q2	430	0	171.66 (165.67, 177.65)		
	Q3	431	0.3	162.41 (156.65, 168.18)		
	Q4	430	1.4	168.11 (162.00, 174.21)	0.74	0.86

Abbreviation: VAT, visceral adipose tissue.

^a^ Multivariable‐adjusted mean VAT areas (cm^2^) are presented for participants categorized into quartiles based on their reported dietary intake. Models were adjusted for age (continuous), sex (male [ref], female), racial and ethnic groups (White [ref], African American, Native Hawaiian, Japanese, Latino), total adiposity from whole‐body dual energy X‐ray absorptiometry (continuous) and total energy intake (kilocalories per day, continuous).

^b^ *p*‐trend values (calculated using a variable that was assigned the median value for each quantile) indicate the strength of a linear dose‐response association across quartiles of dietary intake. *Indicates variables with a significant *p*‐trend after Bonferroni correction.

^c^ In the previous literature, both inverse and positive associations were reported between copper intake and VAT.

^d^ Quartiles of coffee and sugar‐sweetened beverages were unevenly distributed because of a higher number of participants reporting no intake of these beverages.

Further sensitivity analyses were conducted by additionally adjusting the multivariable models for total fat mass and similar results were observed for most foods and nutrients (Table S3). Exceptions were that following additional adjustment for total fat mass the *p*‐trends for monounsaturated fatty acids, omega‐3 fatty acids and total fat were attenuated and no longer significant (*p* ≥ 0.08) although the patterns of higher VAT with increasing levels of intake remained.

## 4 DISCUSSION

The current cross‐sectional study adds important new data to the literature to evaluate specific dietary factors that are associated with the propensity to accumulate VAT in the racially and ethnically diverse MEC‐APS population. The current study confirmed previously reported positive associations between higher dietary intake levels of potatoes and fat (total fat and SFAs) on VAT^3^, ^5^, ^8^ and inverse associations between higher dietary intake of total vegetables, total fruits (including juice), cereals, whole grains, dietary fiber and calcium with lower VAT.^3^, ^5^, ^6^ There was also an inverse association between copper intake and VAT in the current study; previous reports have observed both inverse^4^ and positive^7^ associations with copper intake.

Some notable differences between results in the current study as compared with previous reports were that we observed a positive association between higher margarine intake and VAT, whereas van Eekelen et al.^6^ reported an inverse association. There were also positive associations for monounsaturated and omega‐3 fatty acid intake on VAT in the current study, which contrasts with an earlier report based on a small number (*n* = 167) of participants from Jordan that found inverse associations with VAT.^7^ Lastly, there was no association for dietary intake of sugar‐sweetened beverages, alcohol, coffee, protein and omega‐6 fatty acids on VAT in the current study, which contrasts with positive associations (sugar‐sweetened beverages, alcohol) and inverse associations (coffee, protein and omega‐6 fatty acids) with VAT reported in earlier studies.^3^, ^7^

Foods and nutrients that met the multiple comparison‐corrected significance level included inverse associations with total fruit, whole grains, fiber and copper intake on VAT and positive associations between SFA intake and VAT. The observation of inverse associations of higher dietary intake of fruits, vegetables, whole grains and fiber with VAT in the current study is consistent with prior studies.^3^, ^5^, ^6^ A recent study in a Northern German population also observed that a healthy plant‐based diet was associated with lower VAT.^17^ Regular consumption of plant‐based foods is inversely correlated with the risk of developing type 2 diabetes (more pronounced associations have been reported for whole grains and fiber^18^) and all cause mortality (a marker of potential disease reduction).^19^ It is plausible that higher dietary intake of fruits, vegetables, whole grains and fiber may lower VAT because dietary fiber (a component of fruits, vegetables and whole grains) slows gastric emptying and macronutrient absorption from the gut.^20^, ^21^ The consumption of dietary fiber may also lead to more favorable glucose metabolism.^22^ Higher consumption of fruits, vegetables and whole grain products has been linked to a more favorable gut microbiome.^23^ The inverse association between copper and VAT observed in the current study was consistent with one earlier study^4^ but not another.^7^ Sources of copper include organ meats, seafood, nuts, seeds, wheat bran cereals and wholegrain products.^24^ The Recommended Dietary Allowance for copper for adults is 0.9 mg, which was met by most of the MEC‐APS study participants (those in Q2–Q4 of copper intake). The result for copper in this study is in line with the observation that whole grains were also associated with lower VAT.

Results from the current study and earlier reports^3^, ^5^, ^8^ show a mostly consistent positive association between higher intake of total fat and SFAs on VAT. SFAs are often animal‐derived, and the positive association with higher VAT in the current study provides support for a potential health adverse effect of high SFA intake. Since there is ongoing debate over whether lowering SFA consumption should be recommended in public health guidelines,^25^ it is of interest to evaluate associations between SFA consumption and VAT in future prospective and intervention studies. In contrast, it has been hypothesized that higher intake of certain fat subtypes (including monounsaturated fatty acids) may be considered beneficial. Oils that contain monounsaturated fatty acids include canola and olive oil.^24^ However, in this study, there was a positive association for monounsaturated fatty acid intake with VAT. This finding could be due to the differing sources of monounsaturated fatty acids (e.g., intake of animal products [primarily meat fat] versus olive oil) across populations.^26^

The current study had several key strengths; to our knowledge, this was the first study to evaluate associations between specific foods and nutrients in relation to VAT in a racially and ethnically diverse study population across a range of BMI using a comprehensive FFQ for dietary assessment that has been developed and validated for use in the MEC ethnic groups. Total adiposity was measured using DXA and VAT by abdominal MRI, which are gold‐standard methodologies. Limitations of this study include the cross‐sectional design; therefore, the temporality of associations cannot be inferred. Although the study sample size was substantial and larger than almost all previous reports, the modest number of participants by racial and ethnic groups did not allow for stratified analysis by race and ethnicity. The current analysis focused on older adults in their 60s and 70s; many people in this age group experience aging‐associated changes in body composition, specifically a decrease in fat free mass,^27^ and thus these results may not be generalizable to young or middle‐aged adults. Nonetheless, in this study, adjustment for age did not change the diet‐VAT associations, and the adjustment for total adiposity likely achieved estimating the associations of the diet on the VAT proportion.

The participation rate for this study was modest and may have resulted in the selection of healthier participants; however, the study was optimally designed to address the primary research objective to compare the relative size of fat deposits across five racial and ethnic groups that were representative of the full range of overall adiposity values.^9^ Furthermore, the stratified recruitment design of the MEC‐APS allowed for a relatively even representation by sex, race, ethnicity and BMI categories, thus minimizing any unequal bias due to high refusal or ineligibility. Lastly, results were presented as quartiles of reported dietary intake levels of specific foods and nutrients in the MEC‐APS study population; this approach may limit the ability to compare results across different study populations.

In summary, the current study tested whether dietary intake of specific foods and nutrients that had significant associations with VAT in previous reports (in studies representing mostly White participants) were associated with VAT in the racially and ethnically diverse MEC‐APS population. This report confirmed positive associations with potatoes, total fat and SFA and inverse associations with total vegetables, total fruits, cereals, whole grains, dietary fiber, calcium and copper on VAT. Furthermore, associations with SFA, total fruit, whole grains, fiber and copper met the specified threshold for multiple comparison‐corrected significance; these foods and nutrients may be the best candidates for future research on dietary factors and VAT using prospective or intervention trial study designs. Studies on VAT are clinically relevant because higher VAT is an indicator of obesity‐associated metabolic stress and higher VAT levels have been shown to increase the risk of insulin resistance and type 2 diabetes. In the long term, results from this study can assist in developing improved dietary guidelines to lower VAT and contribute to initiatives that aim to prevent the development of a range of chronic diseases.

## AUTHOR CONTRIBUTIONS

Melissa A. Merritt, Carol J. Boushey, Johanna W. Lampe, Tanyaporn Kaenkumchorn, Loïc Le Marchand, Unhee Lim and Lynne R. Wilkens designed the research plan. Carol J. Boushey, Thomas Ernst, Loïc Le Marchand, Unhee Lim, John A. Shepherd and Lynne R. Wilkens conducted the data collection for the Multiethnic Cohort Adiposity Phenotype study. Carol J. Boushey, Unhee Lim and Lynne R. Wilkens provided essential databases needed for the research. Melissa A. Merritt analyzed data and performed statistical analysis. Melissa A. Merritt wrote the paper. Melissa A. Merritt and Loïc Le Marchand had responsibility for final content. All authors (Melissa A. Merritt, Unhee Lim, Johanna W. Lampe, Tanyaporn Kaenkumchorn, Carol J. Boushey, Lynne R. Wilkens, John A. Shepherd, Thomas Ernst and Loïc Le Marchand) edited the manuscript and approved the final manuscript prior to submission.

## CONFLICT OF INTEREST STATEMENT

Thomas Ernst is a consultant for KinetiCor, Inc, which played no role in this study. The other authors have no conflicts of interest.

## Supporting information

Supporting Information S1

## Data Availability

Data described in the manuscript, code book, and analytic code will be made available from the corresponding author on reasonable request pending approval by the Multiethnic Cohort investigators (see: https://www.uhcancercenter.org/for‐researchers/mec‐data‐sharing).
