# Supplementary material for: Dietary intake and visceral adiposity in older adults: The Multiethnic Cohort Adiposity Phenotype study
Source: Obes Sci Pract. 2024 Jan 22;10(1):e734. doi: 10.1002/osp4.734 (PMC10802887; doi:10.1002/osp4.734)
Supplement: Supplementary file 1 — Supporting Information S1 [file OSP4-10-e734-s001.pdf]

Supplemental materials

Dietary intake and visceral adiposity in older adults: the Multiethnic Cohort Adiposity Phenotype Study

Merritt et al.

Supplemental Methods: description of how foods and nutrients were identified to include in the current study

Firstly, a comprehensive systematic review had been published in 2015 summarizing evidence from human observational and controlled intervention studies that evaluated associations between qualitative aspects of diet and associations with visceral adipose tissue (VAT) and/or subcutaneous adipose tissue (SAT) (1).

Therefore we identified dietary factors that were associated with the propensity to accumulate VAT and for which comparable dietary data were available for analysis in the Multiethnic Cohort (MEC) Adiposity Phenotype Study (APS).

Dietary factors and the direction of associations of dietary factors with VAT reported in Fischer *et al*. (1) are listed. All of the following dietary factors were selected to analyze in the current study, with the exception of phytochemicals.

1. Alcohol : ↑ risk of VAT only (males)
2. Fiber: ↓ risk of VAT only (youths)
3. Total protein: no association with VAT; High protein diet: ↓ risk of VAT
4. Calcium: ↓ risk of VAT
5. Sugar-sweetened beverages increased risk of VAT
6. Phytochemicals (not included in current study): ↓ risk of VAT only (East Asian populations); note: phytochemicals were not assessed in the current study because “phytochemicals” may refer to carotenoids, flavonoids, isoflavones, etc, and looking at the references in Fischer et al they were referring to very small trials in Japan that had examined intake of supplements (e.g., apple polyphenol supplements). The focus of the current study was on diet (foods and nutrient intake) and not supplements therefore phytochemicals were not analyzed in the current report.
7. Low glycemic load diet: ↓ risk of VAT (current study analyzed carbohydrate intake as a proxy)
8. Total fat: ↑ risk of VAT; but it was noted that randomized controlled trials observed no reduction in VAT in individuals who followed a low-fat diet.
9. Whole grain foods: ↓ risk of VAT
10. Refined grain foods: ↑ risk of VAT
11. Coffee: ↓ risk of VAT

To identify foods or nutrients that were associated with VAT in studies that were published after the Fischer *et al*. (1) review, we searched the PubMed database using the search terms "visceral fat" and "diet" with a focus on meta-analyses, randomized controlled studies, prospective cohort studies, large cross-sectional studies and reviews for recent studies that identified dietary factors that were statistically significantly associated with VAT [from September 2013 (when the Fischer *et al.* (1) review period had ended) through to 31 July 2021].

Only studies that had been conducted in humans were eligible for inclusion, studies on diet quality (scores) were excluded and visceral fat had to be estimated using computed tomography or magnetic resonance imaging (MRI) for comparability with the MEC-APS population.

A total of 12 studies were identified that reported on diet and VAT. Of these, eight were excluded because: VAT was not estimated using computed tomography or MRI (N=5); article was no in English (N=2) and article was a thesis only and had not been peer-reviewed (N=1); this left four studies that were used to identify dietary factors to include in the current analysis (**Supplemental Table 1**).

Supplemental Table 1. Studies investigating the association between qualitative aspects of diet and visceral adipose tissue.

Reference	Country	Design and setting	Analytical sample (N)	Characteristics	Diet assessment tool	Dietary aspect analyzed	Covariates/ adjustments	Method and level of VAT assessment	Association with diet (+/-/~)
Rosqvist 2014 (2)	Sweden	Clinical trial: 7 weeks	Thirty-nine young and normal-weight individuals age 20–38 years, BMI 18–27 kg/m2	M,F (young adult)	Fed muffins (overfed)	saturated fatty acids (SFAs) or polyunsaturated fatty acids (PUFAs).		MRI to quantify VAT	VAT (+) SFAs caused a twofold higher increase in VAT than omega (n-6) PUFAs.
Ruttgers 2015 (3)	Germany	Cross sectional	344 men and 241 women (585 total)	M,F (adult)	FFQ	Food groups on FFQ	age, sex, energy intake, physical activity, intake of other food groups and mutual adjustment for VAT and SAAT	MRI to quantify total volume of VAT	VAT (+)<br>Potatoes<br><br>VAT (-)<br>Cake<br>Cereal
Tayyem 2019 (4)	Jordan	Cross-sectional Royal Medical Services personnel	167 healthy adults	M,F (adult)	FFQ	macro- and micronutrients	energy, age, gender, physical activity and smoking	MRI for VAT	VAT (+)<br>Copper<br>Soluble & insoluble fibers<br><br>VAT (-)<br>Total fat<br>Monounsat fat

Reference	Country	Design and setting	Analytical sample (N)	Characteristics	Diet assessment tool	Dietary aspect analyzed	Covariates/ adjustments	Method and level of VAT assessment	Association with diet (+/-/~)
									omega-3 and omega-6
Van Eekelen 2019 (5)	Netherlands Population-based	Cross sectional	6671 participants aged 45–65 y at baseline	M,F (adult)	FFQ	Main food groups	stratified by sex and adjusted for age, smoking, education, ethnicity, physical activity, basal metabolic rate, energy-restricted diet, menopausal state, and total energy intake	MRI for VAT	-Women only: VAT (+) None identified<br><br>VAT (-) Fruit & Vegetable plant-based fats and oils [ie margarine, cooking oils]<br><br>Similar but weaker patterns observed in men

Supplemental Table 2. Associations of intake of total fruits and vegetables assessed as cups/day with the propensity to accumulate VAT[a] in the MEC-APS

Variable	Quartiles of intake	Participants (n)	Median intake	Mean VAT area (95% CLs)	P-trend[b]
Total Vegetables (G)	Q1	430	146.6	172.09 (165.78, 178.40)	0.01
	Q2	430	257.9	167.12 (161.28, 172.96)	
	Q3	431	374.6	170.26 (164.49, 176.03)	
	Q4	430	627.2	158.19 (151.56, 164.82)	
Vegetables (Cup)	Q1	430	0.9	172.53 (166.28, 178.77)	0.01
	Q2	430	1.6	169.46 (163.60, 175.31)	
	Q3	431	2.3	166.64 (160.86, 172.42)	
	Q4	430	3.9	158.95 (152.42, 165.48)	
All Fruits Plus Juice (G)	Q1	430	66.4	172.58 (166.57, 178.58)	<0.001
	Q2	430	163.6	170.62 (164.81, 176.44)	
	Q3	431	285.2	167.04 (161.30, 172.79)	
	Q4	430	532.1	157.54 (151.35, 163.73)	
Fruits, Total, Whole+Juice (Cup)	Q1	430	0.5	174.06 (168.04, 180.08)	<0.001
	Q2	430	1.1	169.44 (163.64, 175.24)	
	Q3	431	1.9	169.20 (163.45, 174.94)	
	Q4	430	3.5	155.00 (148.79, 161.21)	

[a]Multivariable-adjusted mean VAT areas (cm^2) are presented for participants categorized into quartiles based on their reported dietary intake. Models were adjusted for age (continuous), sex (male [ref], female), racial and ethnic group (White [ref], African American, Native Hawaiian, Japanese, Latino), total adiposity from DXA (continuous) and total energy intake (kilocalories per day, continuous).
[b]P-trend values (calculated using a variable that was assigned the median value for each quantile) indicate the strength of a linear dose-response association across quartiles of dietary intake.

Supplemental Table 3. Associations of intake of specific dietary factors with the propensity to accumulate VAT[a] in the MEC-APS

Variable	Quartiles of intake	Participants (n)	Median intake	Mean VAT area (95% CLs)	P-trend[b]	Bonferroni Significant
Dietary Fiber (G)	Q1	430	10.8	173.69 (167.71, 179.66)	1.26E-05	*
	Q2	430	17.1	170.36 (165.03, 175.69)		
	Q3	430	24	163.96 (158.73, 169.20)		
	Q4	431	36.2	152.71 (146.33, 159.09)		
Calcium (Mg)	Q1	430	379.3	172.73 (166.68, 178.79)	0.005	
	Q2	430	592	166.35 (161.03, 171.67)		
	Q3	431	820.7	163.29 (157.99, 168.59)		
	Q4	430	1218.1	157.91 (151.49, 164.33)		
Copper (Mg)	Q1	430	0.8	175.09 (168.85, 181.33)	2.10E-05	*
	Q2	430	1.2	168.86 (163.46, 174.25)		
	Q3	431	1.6	164.96 (159.74, 170.18)		
	Q4	430	2.3	151.51 (144.58, 158.44)		
Protein (G)	Q1	430	39.6	166.35 (159.80, 172.90)	0.82	
	Q2	430	59.5	163.10 (157.63, 168.57)		
	Q3	431	78.7	167.86 (162.63, 173.10)		
	Q4	430	114.1	163.22 (155.75, 170.68)		
Monounsaturated Fatty Acids (G)	Q1	430	13.5	160.98 (154.65, 167.30)	0.10	
	Q2	430	20.9	162.45 (156.97, 167.92)		
	Q3	431	28.6	167.73 (162.51, 172.96)		
	Q4	430	43.8	169.47 (162.32, 176.63)		
Omega-3 Fatty Acids (G)	Q1	430	0.8	161.58 (155.35, 167.80)	0.08	
	Q2	430	1.3	160.65 (155.23, 166.07)		
	Q3	431	1.7	169.00 (163.77, 174.23)		
	Q4	430	2.7	169.34 (162.44, 176.24)		
Omega-6 Fatty Acids (G)	Q1	430	6.9	167.22 (160.97, 173.47)	0.70	
	Q2	430	10.6	165.28 (159.85, 170.71)		
	Q3	431	15	162.77 (157.54, 168.01)		
	Q4	430	22.5	165.29 (158.38, 172.21)		
Total Vegetables (G)	Q1	430	146.6	170.21 (164.49, 175.92)	0.01	
	Q2	430	257.9	165.07 (159.78, 170.37)		
	Q3	431	374.6	167.36 (162.13, 172.59)		
	Q4	430	627.2	157.95 (151.95, 163.95)		
All Fruits Plus Juice (G)	Q1	430	66.4	169.57 (164.13, 175.01)	0.00046	*
	Q2	430	163.6	170.29 (165.03, 175.55)		
	Q3	431	285.2	163.96 (158.75, 169.16)		
	Q4	430	532.1	156.90 (151.30, 162.50)		
Breakfast Cereals (G)	Q1	430	0.8	167.82 (162.52, 173.13)	0.04	
	Q2	430	3.2	168.23 (162.93, 173.52)		
	Q3	431	8	163.91 (158.70, 169.12)		
	Q4	430	22.7	160.69 (155.29, 166.09)		

Variable	Quartiles of intake	Participants (n)	Median intake	Mean VAT area (95% CLs)	P-trend[b]	Bonferroni Significant
Grains, Whole (Oz)	Q1	430	0.4	171.90 (166.48, 177.33)	0.0022	*
	Q2	430	1	164.64 (159.40, 169.87)		
	Q3	431	1.8	165.73 (160.52, 170.93)		
	Q4	430	3.1	158.40 (152.96, 163.85)		
Margarine (G)	Q1	430	0.3	162.77 (157.33, 168.21)	0.02	
	Q2	430	1.1	162.78 (157.55, 168.01)		
	Q3	431	3	164.19 (158.95, 169.42)		
	Q4	430	7	170.78 (165.31, 176.25)		
Total Coffee (G)	Q1	553	0	162.22 (157.58, 166.85)	0.25	
	Q2	344	8.8	165.05 (159.20, 170.90)		
	Q3	407	36.7	166.80 (161.43, 172.16)		
	Q4	417	229.8	167.56 (162.14, 172.98)		
White Potatoes (Cup)	Q1	430	0	160.17 (154.69, 165.64)	0.03	
	Q2	430	0.1	163.38 (158.10, 168.66)		
	Q3	431	0.2	167.63 (162.40, 172.86)		
	Q4	430	0.4	169.35 (163.61, 175.09)		
Carbohydrate (G)	Q1	430	117.8	174.53 (168.10, 180.95)	0.002	*
	Q2	430	177.4	167.46 (162.00, 172.93)		
	Q3	431	233.1	161.43 (156.19, 166.68)		
	Q4	430	342.1	157.03 (149.79, 164.28)		
Total Fat (G)	Q1	430	35.7	162.74 (156.35, 169.13)	0.09	
	Q2	430	54	159.72 (154.24, 165.19)		
	Q3	431	74	167.72 (162.50, 172.94)		
	Q4	430	111.7	170.39 (163.15, 177.63)		
Saturated Fatty Acids (G)	Q1	430	10.7	161.59 (155.35, 167.83)	0.02	
	Q2	430	16.2	161.94 (156.52, 167.35)		
	Q3	431	22.4	162.98 (157.74, 168.22)		
	Q4	430	34.8	174.10 (167.11, 181.09)		
Sugar-Sweetened Beverages (G)	Q1	447	0	168.15 (162.95, 173.35)	0.56	
	Q2	426	3.7	163.30 (158.04, 168.56)		
	Q3	432	16	165.09 (159.89, 170.30)		
	Q4	416	73.9	163.92 (158.52, 169.33)		
Alcoholic Beverages (Drink)	Q1	430	0	163.86 (158.43, 169.29)	0.51	
	Q2	430	0	167.58 (162.14, 173.02)		
	Q3	431	0.3	162.08 (156.86, 167.31)		
	Q4	430	1.4	167.21 (161.68, 172.74)		

[a]Multivariable-adjusted mean VAT areas (cm^2) are presented for participants categorized into quartiles based on their reported dietary intake. Models were adjusted for age (continuous), sex (male [ref], female), racial and ethnic group (White [ref], African American, Native Hawaiian, Japanese, Latino), total adiposity from DXA (continuous), total energy intake (kilocalories per day, continuous) and total fat mass (kg, continuous).
[b]P-trend values (calculated using a variable that was assigned the median value for each quantile) indicate the strength of a linear dose-response association across quartiles of dietary intake.

Supplemental Figure 1. Inclusion flowchart for MEC-APS dietary intake and visceral adipose tissue study.

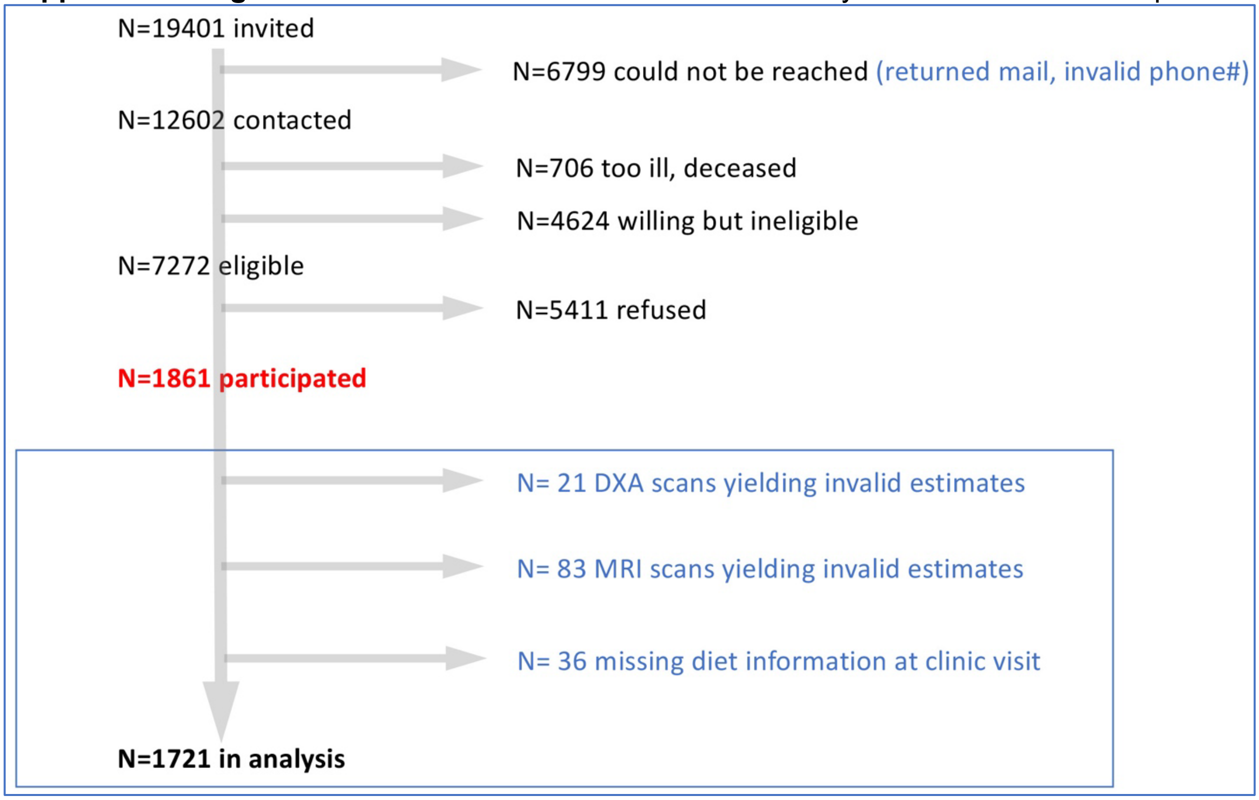

References

1. Fischer K, Pick JA, Moewes D, Nothlings U. Qualitative aspects of diet affecting visceral and subcutaneous abdominal adipose tissue: a systematic review of observational and controlled intervention studies. Nutrition reviews. 2015;73(4):191-215.
2. Rosqvist F, Iggman D, Kullberg J, Cedernaes J, Johansson HE, Larsson A, et al. Overfeeding polyunsaturated and saturated fat causes distinct effects on liver and visceral fat accumulation in humans. Diabetes. 2014;63(7):2356-68.
3. Rüttgers D, Fischer K, Koch M, Lieb W, Müller HP, Jacobs G, et al. Association of food consumption with total volumes of visceral and subcutaneous abdominal adipose tissue in a Northern German population. Br J Nutr. 2015;114(11):1929-40.
4. Tayyem RF, Al-Radaideh AM, Hammad SS, Al-Hajaj S, Allehdan SS, Agraib LM, et al. Subcutaneous and visceral fat volumes measured by MRI and their relationships with nutrient intakes among adults. Asia Pac J Clin Nutr. 2019;28(2):300-9.
5. van Eekelen E, Geelen A, Alssema M, Lamb HJ, de Roos A, Rosendaal FR, et al. Sweet Snacks Are Positively and Fruits and Vegetables Are Negatively Associated with Visceral or Liver Fat Content in Middle-Aged Men and Women. J Nutr. 2019;149(2):304-13.
